# Editorial to the Special Issue “Electrophysiology”

**DOI:** 10.3390/ijms22062956

**Published:** 2021-03-15

**Authors:** Sheng-Nan Wu, Chin-Wei Huang

**Affiliations:** 1 Department of Physiology, College of Medicine, National Cheng Kung University, Tainan 70101, Taiwan; 2 Department of Neurology, National Cheng Kung University Hospital, College of Medicine, National Cheng Kung University, Tainan 70101, Taiwan; huangcw@mail.ncku.edu.tw

Ion channels are well recognized to select ions to pass through the cell membrane in a wide variety of cells. These different types of ion channels are capable of acting to modulate the activities of Na^+^, Ca^2+^, and K^+^ channels in controlling cell excitability. Moreover, cellular electrophysiological studies have indicated that different ion channels, such as hyperpolarization-activated cyclic nucleotide-gated (HCN) channels or voltage-gated K^+^ (K_V_) channels, are essential for various cell functions, such as seizure or pain sensation. Recent emerging progress in the pharmacological characterization of ion channels modulated by different compounds has shown the fundamental importance of ion channels in physiology, pharmacology, and various disorders. It is hoped that this Special Issue will provide researchers of this important and exciting field glimpses into a number of interesting areas worthy of their further studies.

Oxaliplatin (OXAL), a platinum-based anti-neoplastic agent, can increase the amplitude and activation rate constant of hyperpolarization-activated cation current (*I*_h_) with an EC_50_ value of 3.2 µM. This agent resulted in an 11-mV rightward shift in *I*_h_ activation. It also caused an increase in the area of the voltage-dependent hysteresis of *I*_h_. The amplitude of membrane electroporation-induced current was also enhanced by OXAL. Therefore, the OXAL-induced increase in *I*_h_ and *I*_MEP_ may coincide and then act to increase membrane excitability [1].

Dexmedetomidine (DEX) is a highly selective agonist of a_2_-adrenergic receptors. However, a recent study demonstrated its ability to perturb *I*_h_ with an IC_50_ value of 1.21 µM. The voltage hysteresis of *I*_h_ in response to a long triangular ramp pulse was concentration-dependently reduced during exposure to DEX. The results reflect that during exposure to DEX used at clinically relevant concentrations, the DEX-mediated block of *I*_h_ tends to be direct and may be one of the ionic mechanisms underlying decreased membrane excitability [2].

Croton is an extensive flowering plant genus in the spurge family, Euphorbiacea. A recent study demonstrated that three croton compounds with the common *ent*-kaurane skeleton, which was purified from *Croton tonkinensis*, produced effective modifications on the amplitude and gating of *I*_h_ in pituitary tumor (GH_3_) and rat insulin-secreting (INS-1) cells. The hysteretic strength of *I*_h_ was also diminished by these compounds [3].

Honokiol (HNK) is a dimer of allylphenol obtained from the bark of Magnolia officinalis. It has been recently reported to perturb strength of different ionic currents. HNK can modify the amplitude, gating, and hysteresis of *I*_h_ in pituitary GH_3_ cells. The IC_50_ value required for its inhibition of *I*_h_ or delayed-rectifier K^+^ current was estimated to be 2.1 or 6.8 µM, respectively. HNK, or its structurally similar compounds, could exercise the pharmacological actions through its perturbations on ionic currents [4].

A recent study demonstrated that, according to electrophysiological measurements from different rat models with atrial fibrillation, nicotinamide phosphoribosyltransferase (Nampt)/nicotinamide adenine dinucleotide (NAD) activity appears to be an important therapeutic target for the genesis of high-fat-induced atrial fibrillation [5].

Ribociclib (RIB) is an orally administered inhibitor of the cyclin-dependent kinase-4/6 (CDK-4/6) complex. However, the addition of RIB was noted to decrease the peak amplitude of *erg*-mediated K^+^ currents along with a slowed deactivation rate of the current in pituitary GH_3_ cells. Its presence also differentially inhibited the peak and sustained components of delayed rectifier K^+^ currents in these cells. RIB-mediated perturbations on ionic currents tend to be upstream of its suppressive action on cytosolic CDK-4/6 activities [6].

OD-1, a scorpion toxin, has been demonstrated to produce a concentration-, time-, and state-dependent rise in the peak amplitude of voltage-gated Na^+^ currents in mHippoE-14 hippocampal neurons. An intrahippocampal injection of OD-1 was observed to generate a higher frequency of spontaneous seizures and epileptiform discharge in lithium–pilocarpine- or kainic acid-induced epilepsy. The OD-1-induced modifications of *I*_Na_ could thus serve as a novel seizure and excitotoxicity model [7].

The in silico assessments from a recent study unveiled that Pitx-2-induced remodeling increased maximum upstroke velocity and decreased the duration of cardiac action potential, conduction velocity, and wavelength, and that disopyramide appears to be effective against Pitx2-induced atrial fibrillation by prolonging the wavelength [8].

Sphingosine-1-phosphate (S1P) is known to be a signaling sphingolipid that acts as a bioactive lipid mediator. Of interest, S1P had multiplex effects in perturbing the large-conductance Ca^2+^-activated (BK_Ca_) channels in catecholamine-secreting chromaffin cells. It has been demonstrated that S1P-mediated stimulation of Ca^2+^-activated K^+^ currents resulted from the elevated cytosolic Ca^2+^, while the inhibition of BK_Ca_ channel activity by S1P appears to be direct [9].
